# Re-defining wearable robots: a multidisciplinary approach towards a unified terminology

**DOI:** 10.1186/s12984-023-01269-7

**Published:** 2023-11-07

**Authors:** Stefano Massardi, Kristín Briem, Jan F. Veneman, Diego Torricelli, Juan C. Moreno

**Affiliations:** 1 Cajal Institute, Spanish National Research Council (CSIC), Madrid, Spain; 2 Department of Mechanical and Industrial Engineering (DIMI), University of Brescia, Brescia, Italy; 3 Centre of Movement Science, Faculty of Medicine, University of Iceland, Reykjavik, Iceland; 4 Hocoma, Volketswil, Switzerland

**Keywords:** Wearable robots, Biomechanics, Engineering, Motor control, Physical therapy

## Abstract

Effective communication is especially important in the wearable robots (WRs) community, which encloses a great variety of devices across different application domains, e.g., healthcare, occupational, and consumer. In this paper we present a vocabulary of terms with the aim to create a common understanding of terms and concepts among the different fields of expertise relevant in the WRs community. Our goal is to develop shared documentation that could serve as a reference to facilitate the use of accepted definitions in the field. The presented vocabulary is the result of different focus group discussions among experts in the field. The resulting document was then validated by presenting it to the WR community through an online survey. The results of the survey highlight a strong agreement in terms of acceptance of the vocabulary, its usefulness, and applicability of the proposed definitions as well as an overall appreciation for its purpose and target. This work represents a pilot study providing unique material for the WR community, encouraging the use of shared agreed definitions. The reported version of the vocabulary has been made available as a live document in a github repository, for public commenting and further improvements.

**Supplementary Information:**

The online version contains supplementary material available at 10.1186/s12984-023-01269-7.

## Introduction

Wearable robots (WRs), such as exoskeletons and soft exosuits, are mechanical or mechatronic devices attached to the human body for augmenting, assisting, or substituting motor functions [1]. WRs can be used in a wide range of applications, spanning from healthcare to industrial use and personal care. In the last decade, many solutions have moved out of the labs into real-world scenarios, characterized by a multitude of functional goals and a diversity of end-users [2, 3]. Due to the multidisciplinary nature of WRs, most of the terminology used in this field has been taken from different backgrounds, such as biomechanics, engineering, motor control, and physical therapy. This can result in imprecise terminology and terms with multiple or context-dependent meanings, leading to inefficient communication between the different actors.

Recently, a survey study collected experts' opinions on the importance of a user-centered approach in WR development [4]. The results highlighted how standardized frameworks on testing methods and design approaches in WRs are missing and how such frameworks should include a more interdisciplinary approach in addressing the needs of the iteration process, especially when safety is concerned.

Another work proposed an innovative online tool including a glossary of attributes related to wearable robot usability (https://www.usabilitytoolbox.ch/glossary). Although specifically focused on usability, the work confirms the importance of promoting accessibility to specific terminology and sharing how different attributes can be interpreted and defined. Creating unambiguous definitions is also an important aspect of generating safety and performance standards. Those currently in development by the American Society for Testing and Materials (ASTM) Committee F48: Formation and Standards for Industrial Exoskeletons and Exosuits include the ASTM F3323-21, Standard Terminology for Exoskeletons and Exosuits. In this context, terms are defined for manufacturers and regulators to understand which devices are in scope, and how certain tests, procedures or requirements must be interpreted. This implies that this terminology is not developed for supporting the research community or the wider audience, and moreover, most standards must be purchased to be accessible. Terminology developed in a standardization context is often too limited in scope and with too narrow a focus to properly support the research and user communities in their interdisciplinary communication. Obviously, it is helpful if different communities developing terminology in a specific domain are aware of ongoing efforts and take those into account. Still, it would be beneficial for both contexts if an adequate and accurate vocabulary is developed in the academic and user community so that terminology adopted for use in standardization is built on a clear basis, and does not introduce confusion or inadequate regulation.

From 2017 to 2021, COST Action CA16116 Wearable Robots, or "Wearable Robots for Augmentation, Assistance or Substitution of Human Motor Functions" WRs (https://www.cost.eu/actions/CA16116/), has supported a targeted discussion on this topic named “Vocabulary project”. The goal of this project was to engage a interdisciplinary team in a structured process to foster a common understanding of terms and concepts across the different fields of expertise. The expected outcome of this project was to construct a live document/repository of recommended terms to help provide a uniform approach to terminology and notation among different backgrounds.

Our focus was to resolve or acknowledge terms, both technical and non-technical, that have conflicting understanding, usage, or definitions between different areas of expertise, including ethical, legal, and societal contexts. The ambition of this vocabulary is to serve as a practical guide for developers, researchers, end-users, and any other type of stakeholder in the field of WRs.

## Methods

A search across over 300 published articles relevant to WR was conducted, and a script identified 146 words that had two or more occurrences within each publication. These were scanned for relevance and any term that was deemed as ‘common knowledge’ or ‘irrelevant’ for the COST Action (consensus of two readers) was removed, which left 90 terms.

**Fig. 1:**
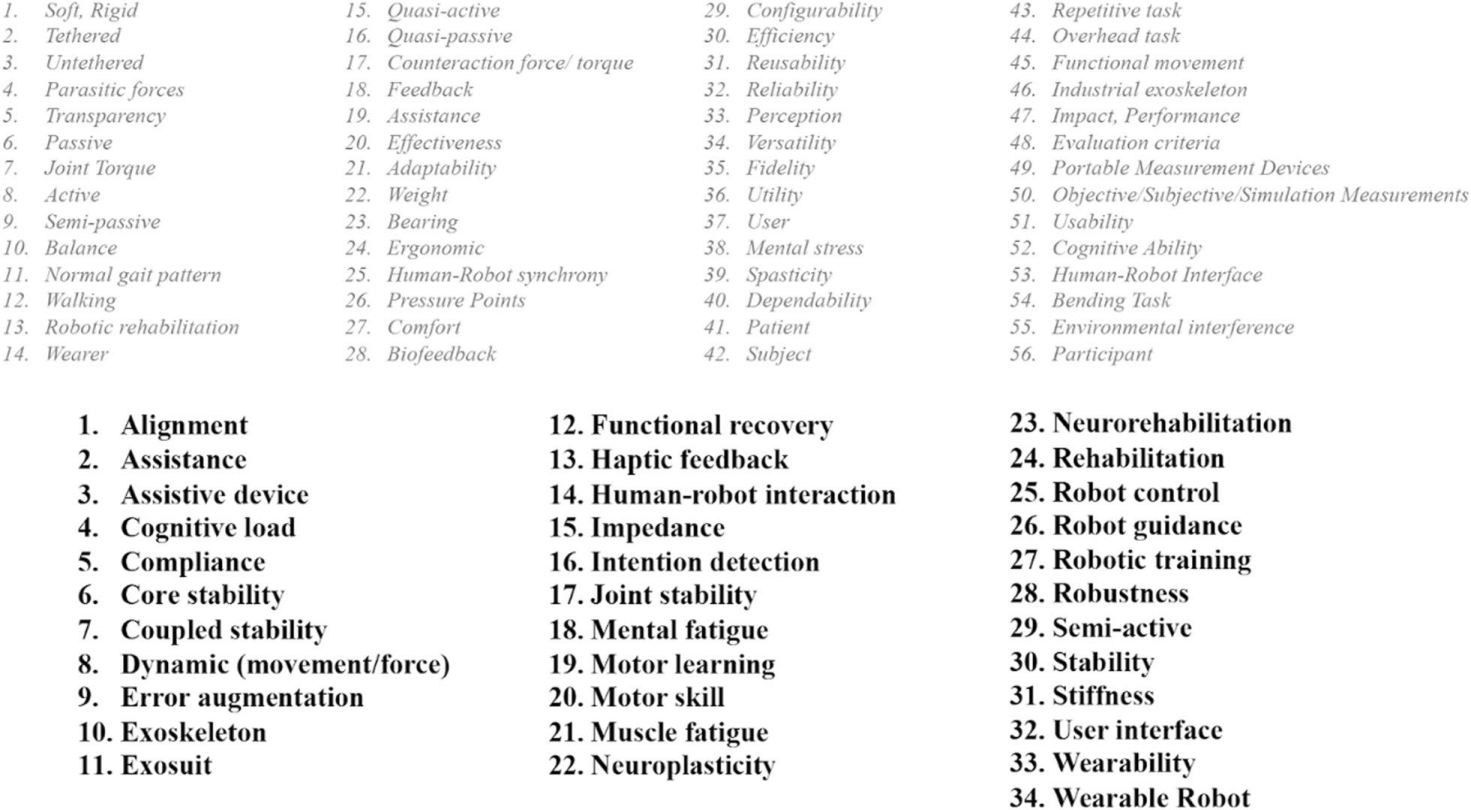
The 90 terms result of the relevance scanning (terms in grey + terms in bold). The terms were further reviewed by the experts who removed 56 terms (grey terms) and selected 34 terms (bold terms) for their further discussion and inclusion in the vocabulary

Sixteen members within COST Action CA16116, as well as external experts, were invited to participate in different focus groups. Each focus group was composed of 3–4 experts with any of the following backgrounds: (i) engineering/technology, (ii) medical/clinical/rehabilitation, (iii) human movement/biomechanics, (iv) ergonomy/human factors, (v) ethical/legal/societal. Some overlap between categories and expert groups was allowed to ensure debate regarding the interpretation and definition of specific terms based on the background knowledge from each discipline. The 90 terms resulting from the relevance scanning were reviewed by all the experts who, through voting, selected those considered most relevant, trying to avoid any overlap or repetitions while ensuring to span different topics. This second review resulted in 34 terms (bold terms in Fig. 1). The 34 terms were further divided and assigned to each focus group that was asked to define them, prioritizing the terms considered most relevant. Each focus group elaborated a set of 3–4 terms producing the first set of definitions. Each term could have multiple definitions. The goal was to reach a consensus on a common definition or a set of different context-dependent definitions for each selected term. The groups of terms were first identified during 2 meetings held in Zurich (2018) and Madrid (2019). The work was also structured inside these workshops and then performed online by the group's components (Additional file 1: https://github.com/WR-Community/WR-Vocabulary).

**Fig. 2:**
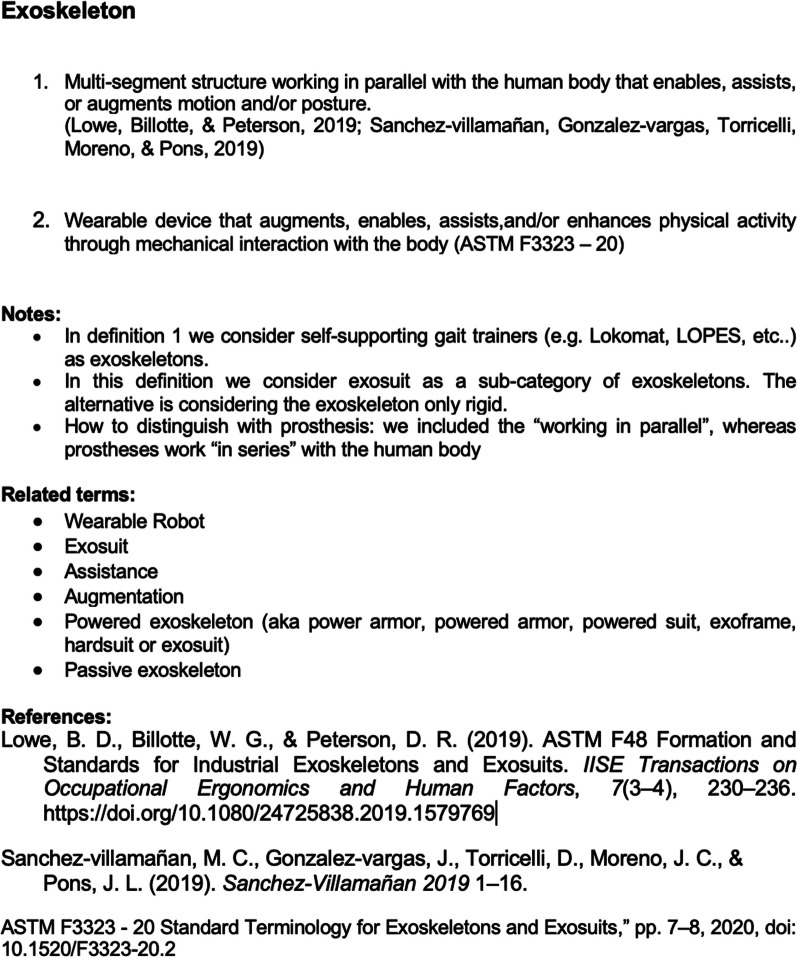
Structure of a single term included in the vocabulary

The focus group participants were asked to analyze existing definitions from academic papers, technical reports, and standardization documents, and decide whether one or more definitions could feasibly apply across disciplines. If no agreement was reached on existing definitions, participants were asked to elaborate one or more new definitions. A third meeting held in Berlin (2019) went through the results of the work done. The 34 terms discussed (highlighted in Fig. 1) are included in a “vocabulary of terms” together with their definitions (see Annex or the GitHub link provided in the “Discussion”), following a page-like structure including the defined term and a list of applicable definitions (one or more), depending on the field. Depending on the discussion that generated the listed definitions, the vocabulary page considers additional fields like (i) a list of relevant notes, (ii) a space for related terms, (iii) a reference section. An example of a page including all the fields is shown in Fig. 2. The agreed definitions could be reached based on group members’ experience and/or a selected literature composed of scientific publications, websites, and international standards listed in Table 1.

**Table 1:** Reference material used for the vocabulary definitions

Publications (complete reference is available in the bibliography section)
Zahedi et al. [5] Alignment of lower-limb prostheses (1986)
Schiraldi et al. [6] Mechanical and kinematic alignment in total knee arthroplasty (2016)
Kibler et al. [7] The role of core stability in athletic function (2006)
Fasse et al. [8] Stability robustness of impedance-controlled manipulators coupled to passive environments (1987)
Liu et al. [9] The effects of error-augmentation versus error-reduction paradigms in robotic therapy to enhance upper extremity performance and recovery post-stroke: a systematic review (2018)
Carmeli et al. [10] Error Augmentation: The Alternative Approach to Treat Brain Injury (2016)
Wei et al. [11] A Real-Time Haptic/Graphic Demonstration of how Error Augmentation can Enhance Learning (2005)
Lowe et al. [12] ASTM F48 Formation and Standards for Industrial Exoskeletons and Exosuits (2019)
Sanchez-Villamañan et al. [13] Compliant Lower Limb Exoskeletons (2019)
Park et al. [14] Design and control of a bio-inspired soft wearable robotic device for ankle–foot rehabilitation (2014)
Belda-Lois et al. [15] Rehabilitation of gait after stroke: a top down approach (2011)
Von Bernhardi et al. [16] What is neural plasticity? (2017)
Khan et al. [17] Neurorehabilitation: applied neuroplasticity (2017)
Moreno et al. [18] Effects of robotic guidance on the coordination of locomotion (2013)
Pollock et al. [19] Clinical Rehabilitation (2000)
Committee et al. [20] International Vocabulary of Metrology, Fourth edition (2021)
Sinkjaer et al. [21] Muscle stiffness in human ankle dorsiflexors: Intrinsic and reflex components (1988)
Gemperle et al. [22] Design for wearability (1998)
Marcora et al. [23] Mental fatigue impairs physical performance in humans (2009)
Gandevia et al. [24] Spinal and supraspinal factors in human muscle fatigue (2001)
Hogan et al. [25] Adaptive Control of Mechanical Impedance by Coactivation of Antagonist Muscles (1984)
Standards
ISO 13482:2014 Robots and robotic devices—Safety requirements for personal care robots
ISO 8373:2012 Robots and robotic devices—Vocabulary
ISO 9241-940:2017 Ergonomics of human-system interaction—Part 940: Evaluation of tactile and haptic interactions
ISO 14593—Ultimate Aerobic Biodegradation
IEC 80601-2-78:2019 Medical electrical equipment—Part 2–78: Particular requirements for basic safety and essential performance of medical robots for rehabilitationassessment, compensation or alleviation
ASTM F3323-21 Standard Terminology for Exoskeletons and Exosuits
Web references
https://en.wikipedia.org/wiki/Fatigue#Mental_fatigue
https://en.wikipedia.org/wiki/Motor_skill
https://en.wikipedia.org/wiki/Muscle_fatigue
https://en.wikipedia.org/wiki/Impedance_control
https://www.who.int/news-room/fact-sheets/detail/rehabilitation

After achieving a consensus on the discussed terms, the vocabulary was presented to the community through a survey. The principal aim of the survey was to collect feedback from the survey participants about the proposed definitions and the structure of the vocabulary. No specific template was used for the creation of this survey. For each of the statements included in the survey, participants were asked to respond on a 5-level Likert scale:

1. Strongly disagree
2. Disagree
3. Neutral
4. Agree
5. Strongly agree

The survey was structured in five sections:

Section 1. Background

This section collected information about participants’ background, including affiliation type (Academic, Company, Others), function profile (Research and Development, Manufacturer, Professional user, Journalist/Blogger, Salesman, Others), and Primary business application domain (Manufacturing, Engineering, Medical, Legal-ethical, Ergonomics, Military, Sport, Healthcare, Others).

Section 2. Needs

This section inquired about the participants’ perceived necessity to provide a tool such as a vocabulary for research-related definitions. The participants could rate the following questions (Q):

- Q1: I feel comfortable handling definitions/terminologies while talking with people from other domains/fields.
- Q2: When discussing topics related to wearable robots, I sometimes experience misunderstandings that could be solved with clearer definitions for given terms.
- Q3: I experience difficulties including definitions/terminologies in papers or documents.
- Q4: I experience difficulties in interpreting or using some terminology when working with wearable robots.

Section 3. Definitions

In this section, participants were asked to review the definitions of 10 terms, randomly extracted from the entire pool. Participants were asked to review at least 5 out of the 10 proposed terms. If a term had multiple definitions, each definition was separately evaluated. Participants could rate the following statements (S) for each definition:

- S1 (agreement)—I agree with the definition.
- S2 (applicability)—This definition is applicable in my field.
- S3 (utility)—I found this definition useful.

Section 4. Open feedback

Participants had the opportunity to propose comments and remarks for each revised definition encountered.

Section 5. Appreciation

The aim of this section was to determine the level of appreciation of vocabulary. Participants were asked to give overall feedback by rating the following questions:

- Q5: This vocabulary can be useful for the community
- Q6: I like the dictionary layout
- Q7: I would use this vocabulary for my communications

The participants could express their wish in contributing and helping to the vocabulary development by providing an email contact for further feedback and collaboration. Incomplete answers were removed from the results analysis.

## Results

The version of the vocabulary that was shared with the community of stakeholders in the field of WRs included 34 terms. 24 terms included a unique definition, 4 terms included 2 definitions, 5 terms included 3 definitions, and 1 term included 4 definitions. Each definition collected from a minimum of 15 to a maximum of 27 answers. For the list of final definitions see the Annex or the GitHub link provided in the “Discussion”.

In the following, we report the feedback received by the respondents of the survey:

### Background

**Fig. 3:**
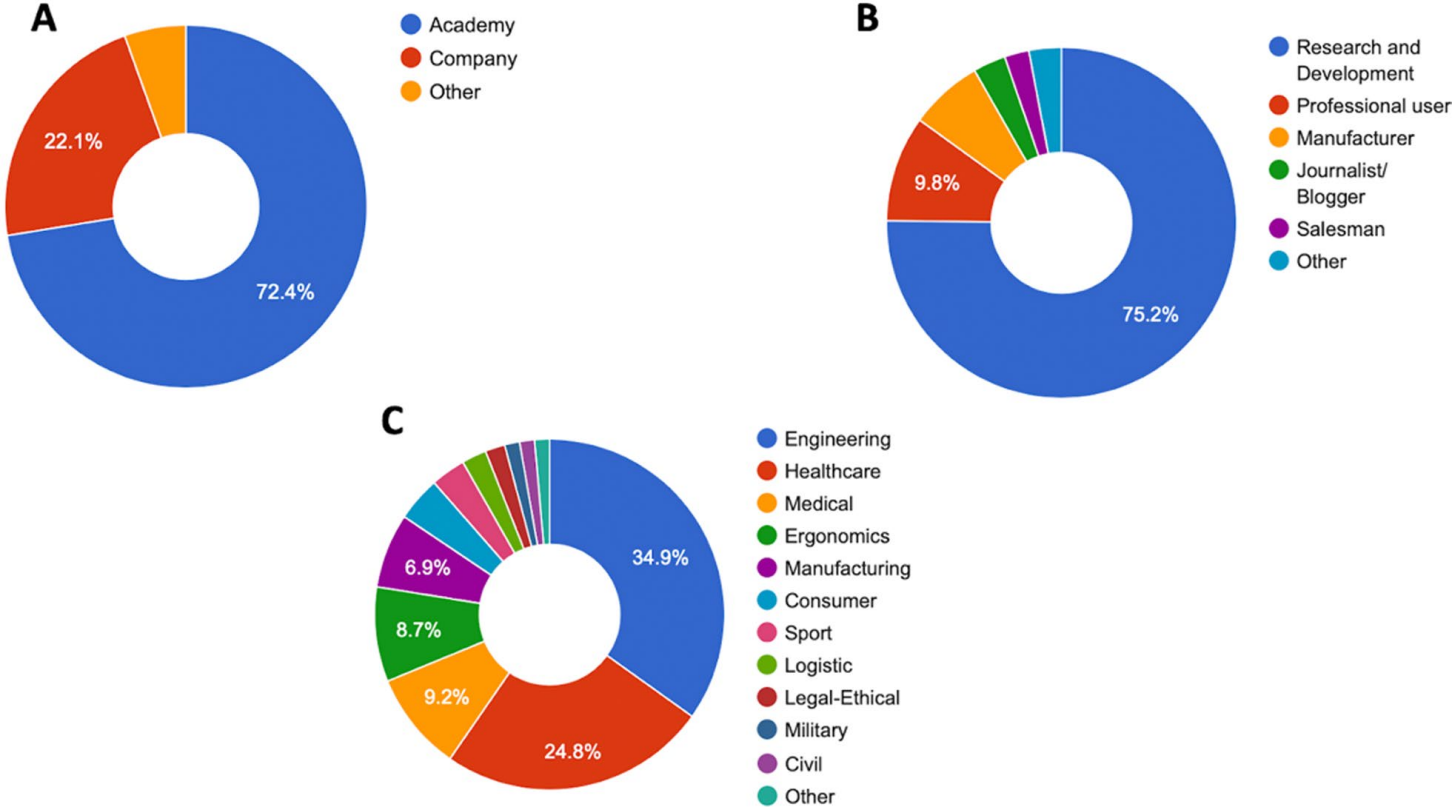
Participant background composed of affiliation type (**A**), function profile (**B**), and Primary business application domain (**C**)

We collected 78 complete answers. Results from section 1 are presented in Fig. 3. Most of the participants’ affiliation type was academic (72.4%) with a research and development profile (75.2%). The main business application domain was Engineering (34.9%) followed by healthcare (24.8%), Medical (9.2%), Manufacturing (6.9%), and other minor fields.

In the following sections, responses are pooled into 3 groups: total agreement (TA), intended as a sum of “strongly agree” and “agree” answers, neutral (N) answers, and total disagreement (TD), as a sum of “strongly disagree” and “disagree” answers.

### Needs

**Fig. 4:**
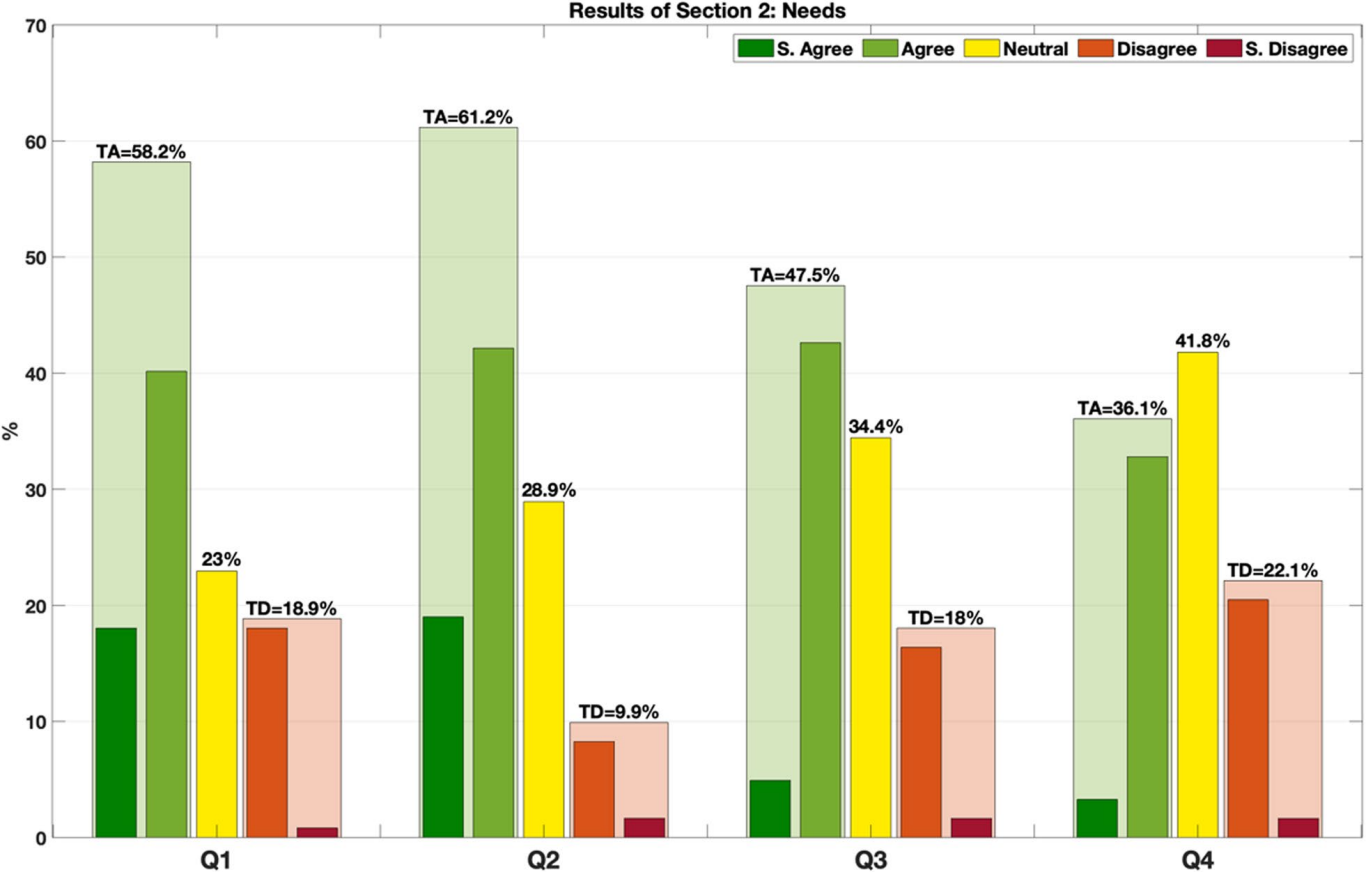
Results of the 4 questions in the “needs” section. Transparent columns in green/red represent the total agreement/disagreement (TA/TD) with the sum of strongly agree/disagree and agree/disagree. Yellow columns represent the remaining neutral answers. Statements: Q1—I feel comfortable handling definitions/terminologies while talking with people from other domains/fields. Q2—When discussing topics related to wearable robots, I sometimes experience misunderstandings that could be solved with clearer definitions for given terms. Q3—I experience difficulties including definitions/terminologies in papers or documents. Q4—I experience difficulties in interpreting or using some terminology when working with wearable robots

Participants generally indicated that they felt comfortable handling definitions/terminologies while talking with people from other domains/fields (58.2% TA, 23% N, 18.9% TD). At the same time, they experienced misunderstandings when discussing topics relating to WRs (61.2% TA, 28,9% N, 9.9%TD). Slightly less than 50% experienced difficulties in including definitions/terminologies in papers or documents (47.5% TA, 34.4% N, 18% TD) or in interpreting or using terminology when working with WRs (36.1% TA, 41.8% N, 22.1% TD). Results of section 2 are presented in Fig. 4.

### Definitions

**Fig. 5:**
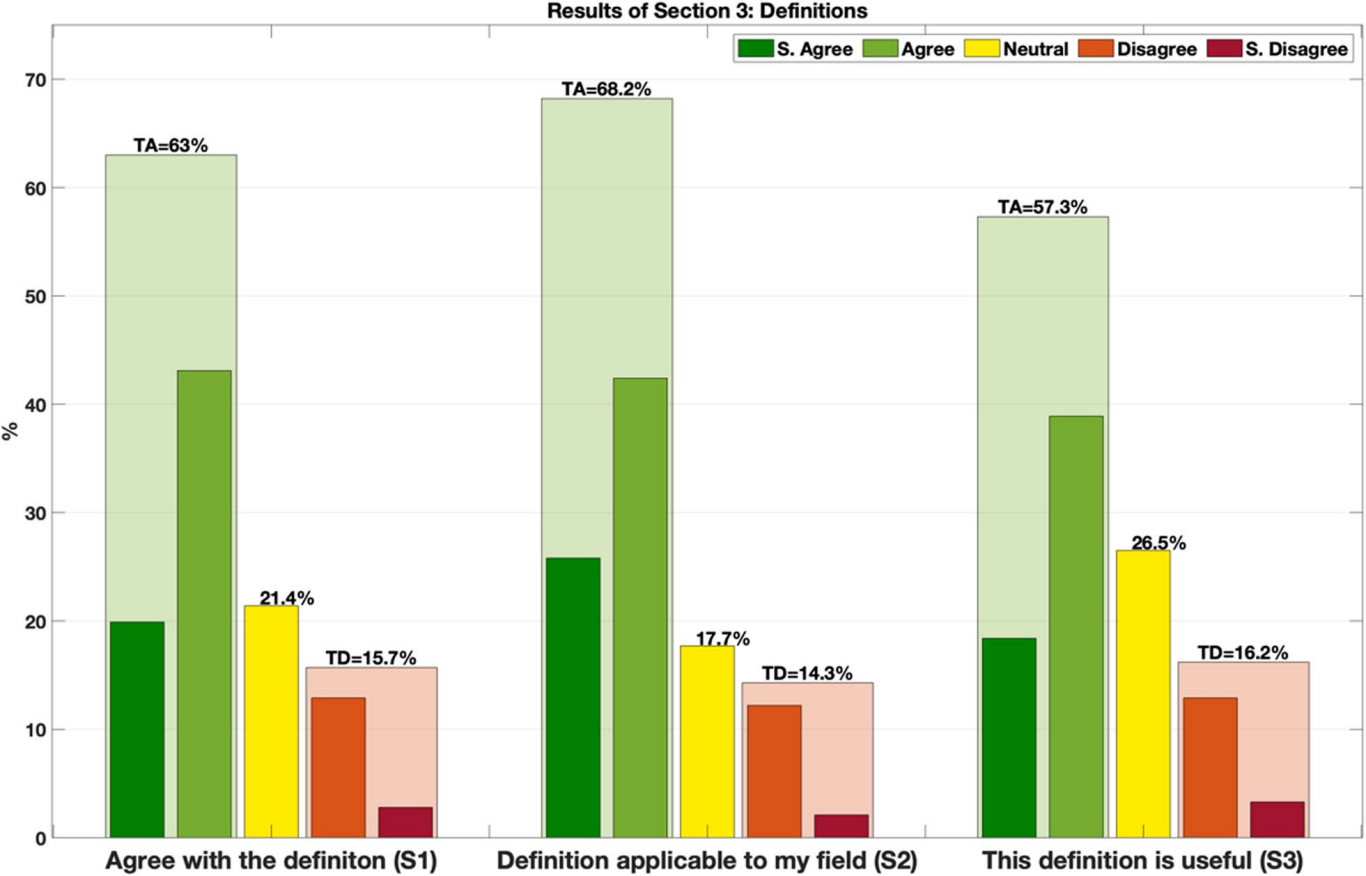
Overall results from the 3 statements proposed for each submitted definition. Transparent columns in green/red represent the total agreement/disagreement (TA/TD) with the sum of strongly agree/disagree and agree/disagree. Yellow columns represent the remaining neutral answers

Survey participants rated the proposed definitions positively in most cases, with 63% of the responses in agreement with the definition (TA), and 21.4% in a neutral position. Definitions were judged by participants to be applicable to their fields in 68.2% of cases (TA), and useful in 57.3% (TA) of the answers. Relatively few answers were found in disagreement. Across the 3 statements, responses indicating TD were 15.7%, 14.3% and 16.2%, respectively. Results of section 3 are presented in Fig. 5.

A mark from 1 to 5 was given to each response: Strongly disagree: 1, Disagree: 2, Neutral: 3, Agree: 4, and Strongly agree: 5. For each definition, three scores were calculated from the average of the marks collected in the submitted statements (average agree score from S1, average applicability score from S2 and average utility score from S3).

**Fig. 6:**
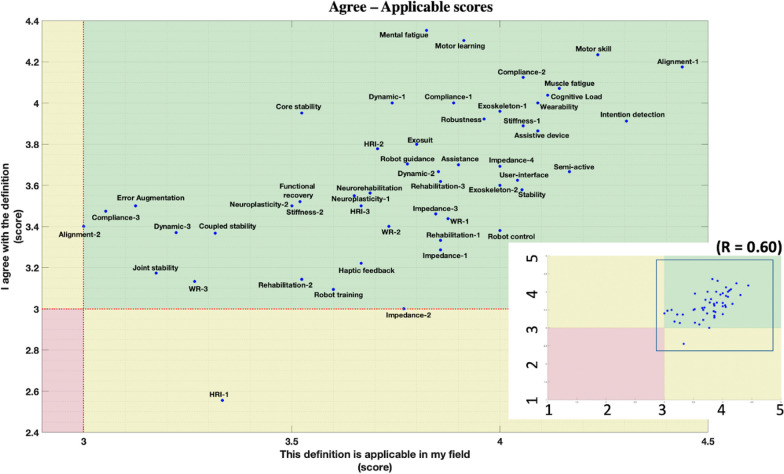
Scatterplot of scores collected from S1 (y-axis) and S2 (x-axis), representing the relation between agreement and applicability of definitions. The full-scale plot is shown at the right-bottom, underlining 3 main areas: green (best, S1 and S2 > 3), yellow (intermediate, S1 or S2 < 3), and red (worst, S1 and S2 < 3). R-value indicates the correlation factor between the S1 score and the S2 score. The main figure zooms out the selection in the right bottom figure. The following abbreviations are applied in the figure: Human–robot-interaction: HRI, Wearable robot: WR. A progressive number is added to the terms including more definitions consistently with the definition number presented in the vocabulary document, e.g., “Dynamic-1” and “Dynamic-2” represent the two definitions of the term “Dynamic”

**Fig. 7:**
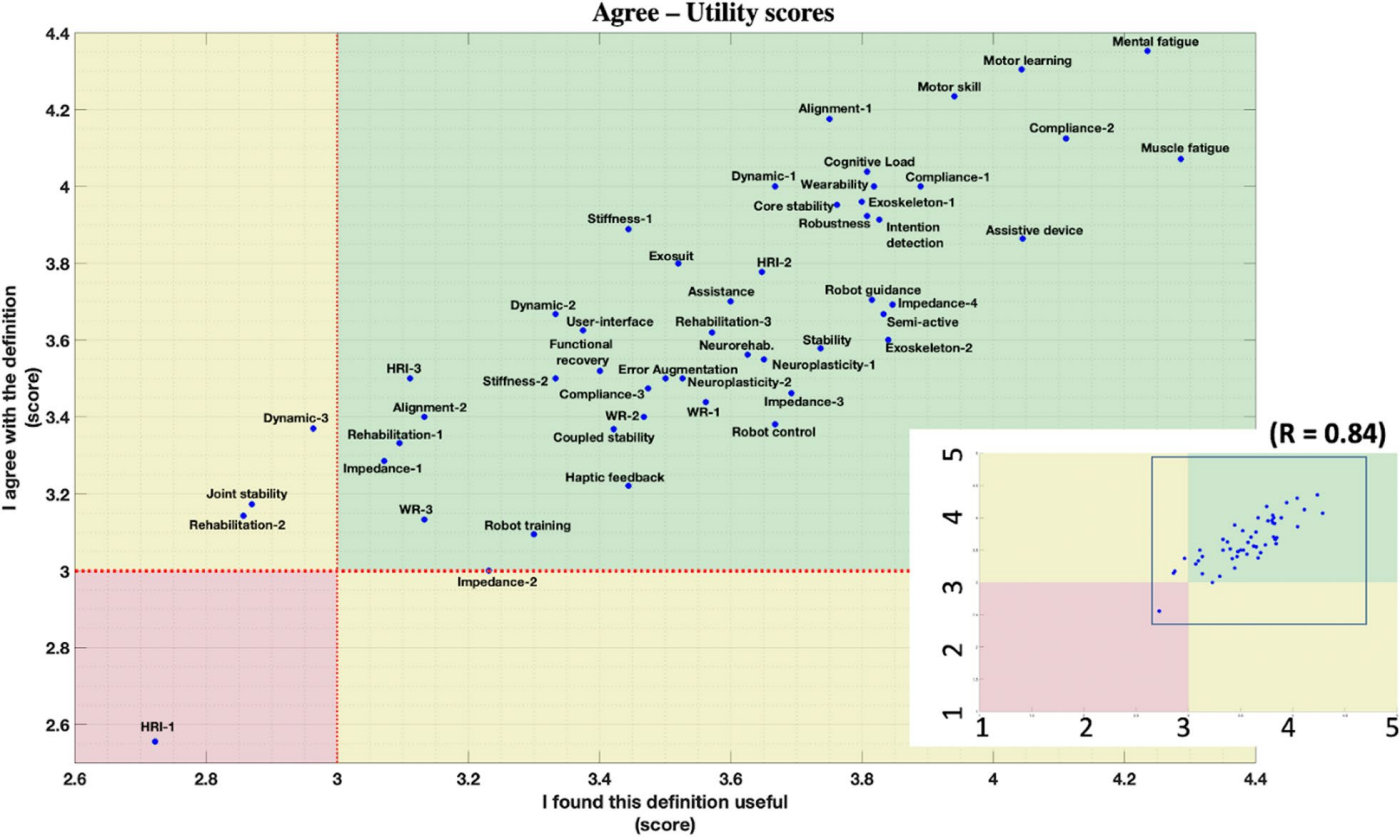
Scatterplot of scores collected from S1 (y-axis) and S3 (x-axis), representing the relation between agreement and applicability of definitions. The full-scale plot is shown at the right-bottom, underlining 3 main areas: green (best, S1 and S3 > 3), yellow (intermediate, S1 or S3 < 3), and red (worst, S1 and S3 < 3). R-value indicates the correlation factor between the S1 score and the S3 score. The main figure zooms out the selection in the right bottom figure. The following abbreviations are applied in the figure: Human–robot-interaction: HRI, Wearable robot: WR. A progressive number is added to the terms including more definitions consistently with the definition number presented in the vocabulary document, e.g., “Dynamic-1” and “Dynamic-2” represent the two definitions of the term “Dynamic”

Average scores collected from the 3 statements—S1 (agreement), S2 (applicability) and S3 (utility)—are represented in scatterplots in Fig. 6 (S1 score on S2 score) and Fig. 7 (S1 score on S3 score) to underline the distribution of the appreciation of single definitions. In these figures, dotted red lines indicate a score of 3 out of 5, which was considered the boundary between negative and positive feedback.

Both figures show that almost all the definitions fall in the positive (green) feedback area. No definitions were found with a score ≤ 3 in S2. Four definitions fell in the yellow area for S3, whereas only one did for S1. “Human–robot interaction” (HRI-1) was the only definition scoring less than 3/5 in both S1 and S3. The definitions falling outside the green area are listed in Table 2.

**Table 2:** Insufficient definition (score < 3)

Statement	Definition	Score
S1: I agree with the definition	Human–robot-interaction-1	2.56
S3: This definition is useful	Dynamic-3	2.96
	Joint stability	2.87
	Human–robot-interaction-1	2.72
	Rehabilitation-2	2.86

Moreover, 10 definitions deviated more than 0.5 score units from the S1–S2 graph (Fig. 6) and the S1–S3 graph (Fig. 7). The maximum score deviation between the dots map was 0.85.

### Open feedback

Respondents commented on 25 terms, resulting in a total of 39 comments. “Stiffness” was the term that received the highest number of comments (five). Three new terms were proposed by participants (“Kinematics”, “Kinetics” and “Physiotherapist”).

In the following we report the comments on the definitions that scored less than 3 out of 5 in at least one of the statements (Table 2):

- The definition for “Rehabilitation” was criticized as too long and confusing. Rehabilitation was considered as a broad concept not necessarily including the use of robotic devices or involving movement.
- One of the respondents suggested that the term “Joint stability” should be defined separately for robots and humans to avoid confusion. Another suggestion was related to its meaning in robotics and control engineering, which should refer to the stability of the control laws. Another comment suggested that “joint stability” should refer to the ability to show a stable response due to external/internal perturbations.
- It was suggested that the term "Dynamic" should be defined closer to the basic definition of physics, as the study of the causes and effects of the movement of objects, i.e., kinematics and kinetics.

The term “Human–robot interaction” collected the lowest feedback, but none of its definitions received comments from the participants.

No comments concerning the vocabulary layout were received.

### Appreciation

**Fig. 8:**
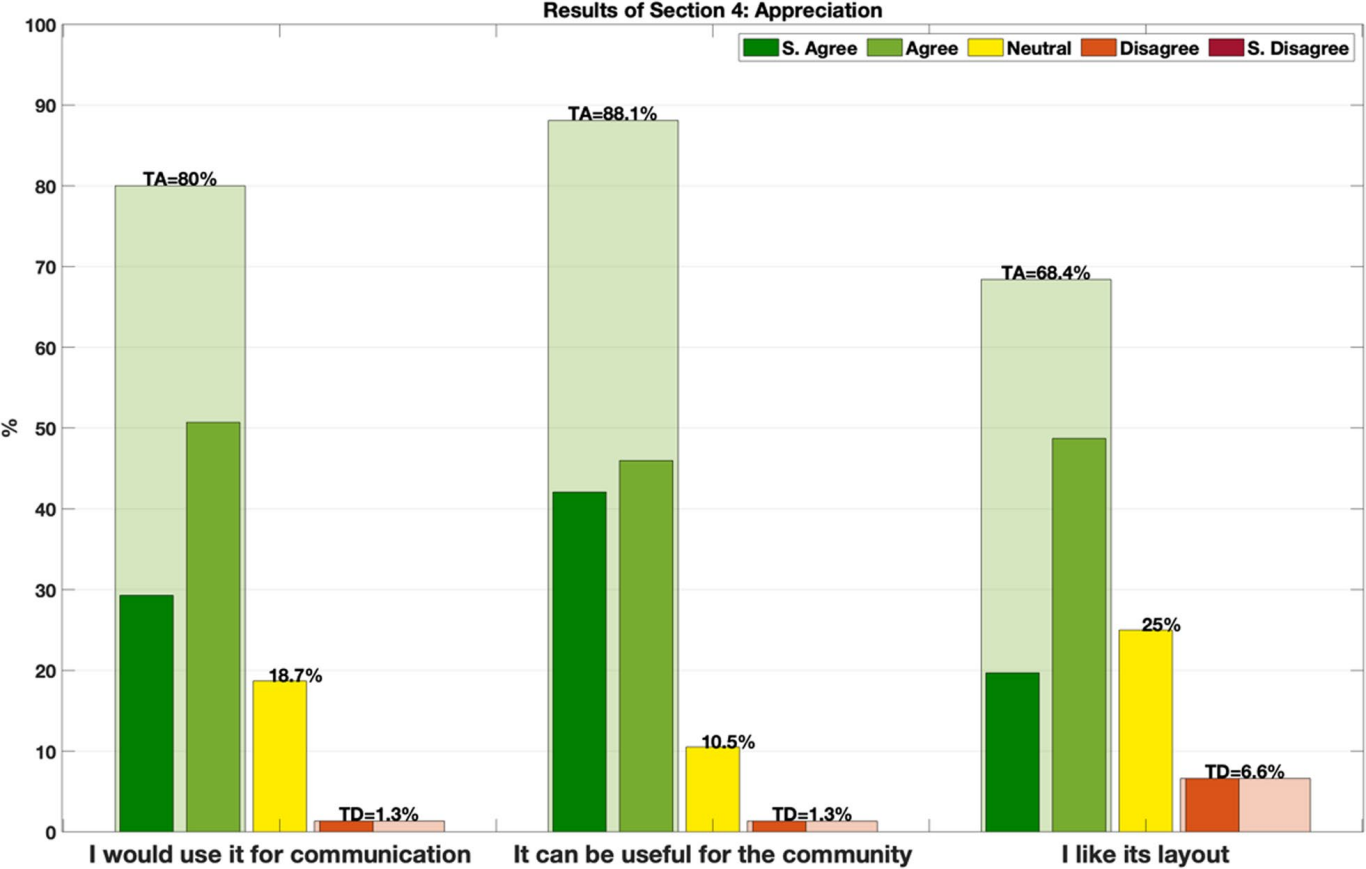
Overall results scored from the 3 questions proposed in the appreciation section. Transparent columns in green/red represent the total agreement/disagreement (TA/TD) with the sum of strongly agree/disagree and agree/disagree. Yellow columns represent neutral answers. The 3 questions refer to the vocabulary presented to the participants asking, “Would you use it for your communication?”, “Do you think it can be useful for the community?” and “Do you like its layout?”

Participants rated the vocabulary project very positively, with 80% (TA) of the respondents indicating that they would use the vocabulary for their communication, whereas 88.2% (TA) of the respondents considered it a useful tool for the community. No responses with strong disagreement were collected in the appreciation questions, with only 1.5% of the responses in disagreement. The Dictionary layout was positively rated in 66.4% (TA) of the answers. Results of section 4 are presented in Fig. 8.

## Discussion

WRs are increasingly available in various configurations (lower limb, upper limb, trunk, spine, low back, active, passive), targeted at different application domains (healthcare, occupational, consumer), and intended for diverse purposes (rehabilitation, augmentation, assistance). This highly heterogeneous field calls for an efficient interaction of an increasing variety of stakeholders, such as developers, end-users, scientists, clinicians, ergonomists, kinesiologists, investors, insurance companies, lawyers, certifying and standardization bodies, policymakers, as well as the general public. When talking about “exoskeletons”, the level of understanding of each of these actors should be harmonized to the specific background and the different levels of technical expertise. It is strongly advisable to have an established terminology able to clearly communicate concepts, goals, needs, and problems. Unfortunately, this is not currently available. The field of WRs is pooling together a plethora of terms from four main areas of knowledge: engineering, medicine, ergonomics, and law. We have observed how a term can be either unknown to other disciplines or have multiple definitions depending on the field. In both cases, a list of terms with concrete definitions is a necessary starting point to involve the different stakeholders in a wider discussion toward international consensus. Our idea of vocabulary does not aim for a standard set of definitions. Instead, it is conceived as a live document to bridge the different disciplines in a common arena of discussion, which may or may not converge into standard definitions. The expected impact is multiple:

1. End-users and society will better understand the functionalities and the potential of WR devices and therefore better accept and trust this new technology.
2. Decision-makers will be able to compare the different technologies available for a specific use-case.
3. Manufacturers will better identify the needs of end-users and communicate their claims for certification/marketing purposes.
4. Experts in regulatory aspects will better identify gaps in standards and modify them to meet market reality.
5. End-users, scientists, and technology developers can better communicate with each other to foster a participatory design strategy to investigate potential hazards and negative consequences of using the technology.
6. Policymakers can more efficiently and rapidly define roadmaps for the introduction and acceptance of rapidly evolving AI-based technologies, bridging ELSE social science with applied science.
7. Professors and trainers can improve the level of training of interdisciplinary professionals, e.g. physiotherapists using robotic technologies, or engineers working in clinical settings.
8. Industrial players and academic researchers can be more tightly connected via technology transfer, common projects, and scientific progress on industry-driven challenges.

The main results of this manuscript are derived from the analysis of the feedback that has been gathered with the proposed survey.

An initial evaluation of this first version of the vocabulary has been completed considering the opinions of focus groups on an initial set of 34 prioritized terms submitted for examination. The findings confirmed the need to have a set of definitions that would be understandable, manageable, and useful across domains that pertain to the field of WRs. The feedback received from the community via online survey demonstrated an overwhelming agreement with several proposed definitions, as well as a positive perception about the rating, acceptance, and usability of most definitions proposed by the expert groups. This study served as an initial proof of concept survey of this interdisciplinary vocabulary as a tool that will likely be appreciated by the WR community, although it requires additional research and follow-up to evaluate its usefulness in the various targeted academic and research domains.

The results of the survey revealed room for improvement of specific definitions and brought to light discrepancies in the way terms are used by disciplines that nonetheless collaborate within the area of WRs. Such conflict in the understanding and use of terms across fields needs to be resolved. Definitions with insufficient agreement rates may have resulted from an unbalanced representation of fields in the focus groups or other characteristics not accounted for in this pilot project. When multiple definitions per term were proposed, the engineering field was preponderant with respect to others less represented, such as medical and biomechanics. Other fields, such as those shown in Fig. 3C, were not specifically represented in the focus groups.

Each respondent was asked to provide a contribution for up to 10 terms, over the 34 included, resulting in a lower number of answers for each definition compared to the total number of respondents. This further division of the data together with the unbalance of the participant's fields (Fig. 3C) did not allow us to conduct a valuable statistical analysis to see whether the agreement or the disagreement collected came from specific areas of expertise or was equally spread.

New terms and definitions were collected, demonstrating an active interest from the respondents to help and contribute to the vocabulary. An interesting trend in the comments received was found in those suggesting an expansion of the proposed definition and to make them more inclusive, and not limited to a specific situation. In contrast, a minor part of the feedback included suggestions to either reduce the spectrum considered or separate the proposed definition into clearer concepts.

Interestingly, established concepts like “Rehabilitation”, “Dynamics” and “Human–Robot Interaction” were criticized, indicating how such general terms may be seen and defined from different points of view.For “Human–Robot Interaction”, the highest score was collected by the standard’s definition (from ISO 8373) whereas the definition proposed by our focus group reached the lowest agreement score, as also happened for the terms “Dynamics” and “Joint stability”. This underlines how some discussed definitions were possibly better defined in the literature and that other factors, such as size and composition of the focus groups, could be a limitation when defining general terms. In addition, some sort of demographic bias among the experts could have led to increasing criticism for some of the proposed definitions, failing to provide a definition applicable to each field.

These factors could explain the gap between scores S1 (definition agreement) and S2 (definition applicability), that was generally more pronounced when compared between S1 and S3 (definition utility). This point draws attention to the need to find consensus on terms considered important but also to consider the field of application and therefore involve different profiles in the discussion to come.

Overall, very few definitions were rated as “insufficient” (score < 3/5), suggesting that the work generally reached a good level of consensus. Furthermore, considering the feedback, we can state that this work raised a notable level of interest from the community. Considering the unbalanced background of the participants (mainly R&D and healthcare/engineering) and the amount of received answers, no further discussions based on the participants background were possible.

Based on these results, we updated the vocabulary targeting the definitions with insufficient acceptance score.

The four terms reported in Table 2 were revised taking into consideration the open comments from the respondents. We proceeded in deleting the “Human–robot interaction” definition, which collected the lowest score and was considered too general and incomplete. The remaining terms of Table 2 were revised and modified. We further reviewed the remaining open comments acting where a need for clarity was detected. Nine additional terms pages were changed after this revision. The actions consisted in either a modification in the term definition or the addition of complementary information in the notes. Moreover, we proceeded to add the three terms proposed by the participants.

However, other terms initially considered (grey terms in Fig. 1) are still without an agreed definition. This, together with greater coverage of all the main backgrounds (lack of medical-oriented definitions) shall be one of the major next improvements foreseen for this vocabulary.

This vocabulary represents an ongoing work and next actions are targeted on its improvement and update. In this direction, an online forum to improve interaction and comments to perform an updated validation of the vocabulary represents an essential step for the desired outcomes. This important step aims to create a platform to actively include the community and compare opinions from different backgrounds. To facilitate the dissemination a continuous update process, the vocabulary document has been added to a public online repository (https://github.com/WR-Community/WR-Vocabulary). This repository will keep the community involved in this endeavor and will ensure a continuous update process based on the collection of opinions and comments. Other minor improvements may be achieved by including diagrams to illustrate concepts, synonyms, and recommended acronyms for each page related to one term.

With further research and open interaction with the community, the vocabulary is expected to evolve, either including more terms in need of clarification. The impact of this work resides in different crucial aspects. Usability and performance tests will need agreed methods, protocols and parameters to ensure a valid and accepted comparison of the results. The recent increasing need of benchmarking efforts for WRs gives rise to the need of harmonized terminology and the need of this type of bibliographic resources, preparing the field for a wider debate between parties from different fields that will have to share common methods and regulations in terms of both performance and safety assessment.

The wearable robot legislation has indeed experienced a recent impulse in the attempt to fill the gap between a technology such as WRs, that is growing faster than its regulation. Confusing and overlapping categories represent an actual issue in this process, especially for devices such as exoskeletons. Also based on the survey results, we are confident that this work could represent an important tool to help an easier inclusion of terminology in manuscripts and documentation preventing misunderstanding and discussion among the different fields embraced by the WR community.

This work represents a valuable starting point for future projects and initiatives that will be able to draw both from the work performed and from the feedback of the community, to produce a more complete and comprehensive tool to support the WRs community in the use of its terminology.

## Conclusions

The findings from this work indicate that there is scope for enhancing definitions and uncovered disparities in the way terms are employed by different disciplines that work together within the domain of WRs.This vocabulary, however, must be considered preliminary. A complete compilation satisfying the entire WRs community, that also includes the opinions of patient end-users, is needed to complete this tool for it to be of assistance in the interpretation of terms within an interdisciplinary context. Moreover, we hold the view that the approach illustrated in this work could have pertinence for other interdisciplinary fields and areas.

### Supplementary Information

**Additional file 1**: The resulting vocabulary document in pdf format is provided in the file Vocabulary Annex.pdf containing the list of terms and definition references in this work. The file is available in the public gitHub directory: https://github.com/WR-Community/WR-Vocabulary.

## Data Availability

Data sharing is not applicable to this article as no new data was created or analyzed in this study.
