# Supplementary material for: Re-defining wearable robots: a multidisciplinary approach towards a unified terminology
Source: J Neuroeng Rehabil. 2023 Nov 7;20:149. doi: 10.1186/s12984-023-01269-7 (PMC10631060; doi:10.1186/s12984-023-01269-7)
Supplement: Supplementary file 1 — Additional file 1: The resulting vocabulary document in pdf format is provided in the file Vocabulary Annex.pdf containing the list of terms and definition references in this work. The file is available in the public gitHub directory: https://github.com/WR-Community/WR-Vocabulary. [file 12984_2023_1269_MOESM1_ESM.pdf]

Re-defining Wearable Robots:
A Multidisciplinary Approach Towards a Unified Terminology

ANNEX

Vocabulary

of terms related to
Wearable Robotics Technologies

Index of terms

Alignment 4

Assistance 5

Assistive device 6

Cognitive Load 7

Compliance 8

Core stability 9

Coupled stability 10

Dynamic 11

Error augmentation 12

Exoskeleton 13

Exosuit 14

Functional recovery 15

Haptic feedback 16

Human-Robot Interaction 17

Impedance 18

Intention detection 19

Joint stability 20

Kinematic 21

Kinetics 22

Mental fatigue 23

Motor learning 24

Motor skill 25

Muscle fatigue 26

Neuroplasticity 27

Neurorehabilitation 28

Physiotherapist 27

Rehabilitation 29

Robot control 31

Robot guidance 32

Robotic training 33

Robustness 34

Semi-Active 35

Stability 36

Stiffness 37

User interface..38
Wearability..39
Wearable robot..40

Alignment

1. Correspondence in position and orientation between the anatomical and robot axes.

2. (Engineering) in human-robotic exoskeleton: rotation and translation axes of the external device in the correct position with respect to the human joint axes.in Human-robotic prosthesis: the position of the socket relative to the other prosthetic components of the limb (Zahedi, Spence, Solomonidis, & Paul, 1986)

3. (Medical) For related concept definitions on anatomical alignment (knee arthroplasty) limb alignment, mechanical alignment refer to: (Schiraldi, Bonzanini, Chirillo, & de Tullio, 2016)

References:

Schiraldi, M., Bonzanini, G., Chirillo, D., & de Tullio, V. (2016). Mechanical and kinematic alignment in total knee arthroplasty. *Annals of Translational Medicine*, *4*(7), 1–5. https://doi.org/10.21037/atm.2016.03.31

Zahedi, M. S., Spence, W. D., Solomonidis, S. E., & Paul, J. P. (1986). Alignment of lower-limb prostheses. *Journal of Rehabilitation R&D*, *23*(2), 2–19.

Assistance

Support a person in achieving a functional goal

Notes:
Assistance can be physical, sensory, cognitive, or a combination of them.

Related terms:

- Support
- Assistive devices

Assistive device

1. product used to support a person with no impairment, generally in activities of worklife, to prevent muscle fatigue, stiffness or other effects of physical workload that could lead to the development of muscle strain or muscoloskeletal disorders.

2. (Medical) Any product (including devices,equipment, instruments, and software), especially produced or generally available, used to aid a person with an injury or disability: (1) for participation in activities of daily living, (2) to protect, support, train, measure, or substitute for body functions/structures and activities, or (3) to prevent impairments, activity limitations, or participation restrictions (ASTM F3323-21)

Notes:
Assistive devices can be used also by healthy subjects

References:
ASTM F3323-21 Standard Terminology for Exoskeletons and Exosuits

Cognitive Load

Information processing effort to complete a task.

Notes:
This definition is applicable to all fields. Examples: level of frustration, sense of security, comfort within your surroundings and interaction with others.

Related terms:

- Mental stress;
- Social Interaction;

Compliance

1. (Engineering) Flexible behaviour of a robot or any associated tool in response to external forces exerted on it. (ISO 8373)
   NOTE When the behaviour is independent of sensory feedback, it is passive compliance; if not, it is active compliance.

2. (Engineering, Biomechanics) Ability to deform in response to external forces, at the level of the application point.

3. (Medical) The degree to which a patient correctly follows medical instructions.

Notes:
Technically the opposite of stiffness.

Related terms:
sensory feedback

References:
ISO 8373 Robots and robotic devices — Vocabulary, vol. 2012. 2008

Core stability

Ability to control the position and motion of the trunk over the pelvis to allow optimum production, transfer and control of force and motion to the terminal segment in integrated athletic activities. (W.B., J., & A., 2006)

Notes:
This definition only covers the aspect concerning the control of the trunk. The term can be used to speak about the core stability of a robot per se, which is how stable a robot is, without any relationship to a body part.

Related terms:
- Stability
- Coupled stability
- Joint stability

References:
W.B., K., J., P., & A., S. (2006). The role of core stability in athletic function. *Sports Medicine (Auckland, N.Z.)*, *36*(3), 189–198.

Coupled stability

Stability of a system composed of robot and environment coupled together mechanically, e.g. wearable robots. A system is said to exhibit coupled stability if:

- the system is stable when isolated;
- the system remains stable when coupled to any passive environment which is also stable when isolated
(Fasse, 1987)

Related terms:

- Stability
- Core stability
- Joint stability

References:

Fasse, E. D. (1987). *Stability robustness of impedance controlled manipulators coupled to passive environments*. Retrieved from https://dspace.mit.edu/handle/1721.1/14805

Dynamic

1. (Medical) Involving motion, as opposed to static. Example: a dynamic vs. isometric exercise.

2. (Biomechanics) Involving forces (synonym of kinetics), complementary to kinematics. Example: inverse dynamics and inverse kinematics.

3. (Engineering, biomechanics). A motion that allows unstable sub-phases, as opposed to quasi-static motion. Example: dynamic walking vs. ZMP.

4. (Physics - General) Study of the causes and effect of motion

Error augmentation

Method to artificially increase the task-specific performance errors.
(Liu, Li, & Lamontagne, 2018)(Wei, Patton, & Scheidt, 2005)

Notes:
It can be used as a rehabilitation method to enhance motor learning by using erroneous sensory feedback to enhance motor adaptation.

Related terms:

- Error augmentation
- Error-driven disturbance
- Sensory feedback
- Performance error

References:

Liu, L. Y., Li, Y., & Lamontagne, A. (2018). The effects of error-augmentation versus error-reduction paradigms in robotic therapy to enhance upper extremity performance and recovery post-stroke: A systematic review. *Journal of NeuroEngineering and Rehabilitation*, *15*(1), 1–25. https://doi.org/10.1186/s12984-018-0408-5

Wei, Y., Patton, J., & Scheidt, R. (2005). *A Real-Time Haptic / Graphic Demonstration of how Error Augmentation can Enhance Learning* *. (April), 4406–4411.

Exoskeleton

1. Multi-segment structure working in parallel with the human body that enables, assists, or augments motion and/or posture.
   (Lowe, Billotte, & Peterson, 2019; Sanchez-villamañan, Gonzalez-vargas, Torricelli, Moreno, & Pons, 2019)

2. Wearable device that augments, enables, assists,and/or enhances physical activity through mechanical interaction with the body (ASTM F3323 – 20)

Notes:

- In definition 1 we consider self-supporting gait trainers (e.g. Lokomat, LOPES, etc..) as exoskeletons.
- In this definition we consider exosuit as a sub-category of exoskeletons. The alternative is considering the exoskeleton only rigid.
- How to distinguish with prosthesis: we included the “working in parallel”, whereas prostheses work “in series” with the human body

Related terms:

- Wearable Robot
- Exosuit
- Assistance
- Augmentation
- Powered exoskeleton (aka power armor, powered armor, powered suit, exoframe, hardsuit or exosuit)
- Passive exoskeleton

References:

Lowe, B. D., Billotte, W. G., & Peterson, D. R. (2019). ASTM F48 Formation and Standards for Industrial Exoskeletons and Exosuits. *IISE Transactions on Occupational Ergonomics and Human Factors*, *7*(3–4), 230–236. https://doi.org/10.1080/24725838.2019.1579769

Sanchez-villamañan, M. C., Gonzalez-vargas, J., Torricelli, D., Moreno, J. C., & Pons, J. L. (2019). *Sanchez-Villamañan 2019* 1–16.

ASTM F3323 - 20 Standard Terminology for Exoskeletons and Exosuits,” pp. 7–8, 2020, doi: 10.1520/F3323-20.2

Exosuit

Exoskeleton made predominantly of soft materials.
(Park et al., 2014)

Notes:
In this definition we consider exosuit as a sub-category of exoskeletons.

Related terms:

- Soft exoskeleton (synonymous)
- Assistance
- Augmentation

References:

Park, Y. L., Chen, B. R., Pérez-Arancibia, N. O., Young, D., Stirling, L., Wood, R. J., … Nagpal, R. (2014). Design and control of a bio-inspired soft wearable robotic device for ankle-foot rehabilitation. *Bioinspiration and Biomimetics*, *9*(1). https://doi.org/10.1088/1748-3182/9/1/016007

Sanchez-villamañan, M. C., Gonzalez-vargas, J., Torricelli, D., Moreno, J. C., & Pons, J. L. (2019). *Sanchez-Villamañan2019_Article_CompliantLowerLimbExoskeletons.pdf*. 1–16.

Functional recovery

Partial or total restoration of a motor function that has been affected due to a lesion at neural or musculoskeletal system, a process involving spontaneous recovery and the effects of therapeutic interventions. (Belda-Lois et al., 2011)

Notes:

- A robot providing a substitution that enables a function. Example: complete SCI patient upper limb movement function is "recovered" thanks to the robot compensation.
- In rehabilitation functional recovery is related to the abilities of the patient and not the recovery of impairments. In this case recovery also involves compensation.

Related terms:
Rehabilitation

References:
Belda-Lois, J.-M., Horno, S. M., Bermejo-bosch, I., Moreno, J. C., Pons, J. L., Farina, D., … Rea, M. (2011). Rehabilitation of gait after stroke:a top down approach. *Journal of NeuroEngineering and Rehabilitation*, *66*(December).

Haptic feedback

1. Activity of sensory receptors in the skin, muscles, joints and/or tendons resulting from physical interaction between the environment and the subject. (ISO 9241-940:2017)

2. Attempt to transfer communication to the user through the sense of touch

Note:
Haptic feedback relates to the human sense of force and pressure.

Related term:
- Haptic interaction
- Physical interaction

References:
ISO 9241-940:2017 Ergonomics of human-system interaction — Part 940: Evaluation of tactile and haptic interactions

Human-Robot Interaction

1. Information and action exchanges between human and robot to perform a task by means of a user interface (ISO 8373)

2. (Human-Machine Interface) The means to realize two types of Human-Robot interaction: physical and cognitive

Notes:
Definition applicable to all fields. Examples of interaction: pressure, force, Joystick, GUI, sensors (EMG, etc).

Related terms:
- Human-Machine/Robot interface
- User interface (related to def. 2)

References:
ISO 8373:2012 Robots and robotic devices — Vocabulary

Impedance

1. (Engineering: Impedance control) Impedance control is an approach to dynamic control relating force and position.
   https://en.wikipedia.org/wiki/Impedance_control

2. (Engineering: Impedance control strategy) It is a general strategy for controlling a manipulator by controlling its motion (as in the conventional robot control) and in addition give it a disturbance response for deviations from that motion which has the form of an impedance.

3. (Engineering: Mechanical Impedance) Relationship between the net force applied on a mechanical system and the system's resulting kinematics, that is, position, velocity, and acceleration.
   (Hogan, 1984)

4. (Biomechanics: Impedance) Hogan's definition: The relation between the neural input to a muscle and its subsequent mechanical behavior.

References:
Hogan, N. (1984). Adaptive Control of Mechanical Impedance by Coactivation of Antagonist Muscles. *IEEE Transactions on Automatic Control*, *29*(8), 681–690. https://doi.org/10.1109/TAC.1984.1103644

Intention detection

Identification of subject's intention to perform a motor task through indicators derived from measured (biological) signals.

Notes:

- "When" and "what" are two aspects that may assume different levels of importance depending on the application (BCI, control, neuromodulation)
- Note that there are many unconscious decisions that are made by the person (i.e. while walking)

Related terms:
Prediction

Joint stability

1. Resistance to movement offered by musculoskeletal tissues around a skeletal joint (to limit the movement to physiological ROM)

2. Ability of a joint to show a stable response to an external or internal perturbation.

Notes:
In robotics and control engineering, joint stability could refer to stability of the control laws that pilot the joint.

Related terms:
Stability
Core stability
Coupled stability

Kinematic

Study of the effect of the movement of objects (Position, Velocity, Acceleration in its linear and angular form).

Kinetics

Study of the causes of the movement of objects (Forces, Moments).

Mental fatigue

Temporary declined cognitive performance under prolonged cognitive load. Not injury related (Marcora, Staiano, & Manning, 2009)
https://en.wikipedia.org/wiki/Fatigue#Mental_fatigue

Notes:
Applicable to engineering, biomechanics and medical indistinctly

Related terms:

- Cognitive performance
- cognitive load
- cognitive capacity

References:

Marcora, S. M., Staiano, W., & Manning, V. (2009). Mental fatigue impairs physical performance in humans. *Journal of Applied Physiology*, *106*(3), 857–864. https://doi.org/10.1152/japplphysiol.91324.2008

Motor learning

The process of acquiring and/or improving a motor skill.

Notes:

- Also used for definition of research fields: Motor learning is a subdiscipline of motor behavior that examines how people acquire motor skills.
- We do not argue with Motor learning on the internal processes involved in skill acquisition.

Related terms:

- Motor adaptation
- Motor recovery
- Motor Skill

Motor skill

Ability to perform a motor task consistently and efficiently across different situations.
https://en.wikipedia.org/wiki/Motor_skill

Notes:
To not be confounded with motor task

Related terms:

- Motor learning
- Motor recovery

Muscle fatigue

Temporary declined ability of muscles to develop force. Not injury related. Reversible by rest. (Gandevia, 2001)
https://en.wikipedia.org/wiki/Muscle_fatigue

Notes:
Applicable to engineering, biomechanics and medical indistinctly.

Related terms:
- Muscle weakness
- Paresis

References:
Gandevia, S. C. (2001). Spinal and supraspinal factors in human muscle fatigue. *Physiological Reviews*, *81*(4), 1725–1789. https://doi.org/10.1152/physrev.2001.81.4.1725

Neuroplasticity

1. Functional and/or structural modification of the nervous system in response to stimuli and/or injury
   (Von Bernhardi, Eugenín-Von Bernhardi, & Eugenín, 2017)

2. Endogenous neural plasticity: neural plasticity that arises from intrinsic or endogenous factors such as spontaneous recovery, experience or the use of cognitive functions.

3. Exogenous neural plasticity: neural plasticity that arises from extrinsic or exogenous factors such as brain stimulation or drug-induced neural plasticity.

Related terms:
- Neurorehabilitation
- Motor learning

References:
Von Bernhardi, R., Eugenín-Von Bernhardi, L., & Eugenín, J. (2017). What is neural plasticity? *Advances in Experimental Medicine and Biology*, *1015*, 1–15. https://doi.org/10.1007/978-3-319-62817-2_1

Neurorehabilitation

A healthcare process aiming to promote recovery from a nervous system injury/disease. (Khan, Amatya, Galea, Gonzenbach, & Kesselring, 2017)

Notes:

- It is an interdisciplinary process requiring coordinated effort of diverse sectors (professions, patients and community).
- Look at RACA (Rehabilitation, Alleviation, Compensation, Assessment): IEC 80601-2-78

Related terms:

- Recovery
- Impairment
- Motor learning
- Neuroplasticity
- Robot-aided neurorehabilitation

References:

Khan, F., Amatya, B., Galea, M. P., Gonzenbach, R., & Kesselring, J. (2017). Neurorehabilitation: applied neuroplasticity. *Journal of Neurology*, *264*(3), 603–615. https://doi.org/10.1007/s00415-016-8307-9

Physiotherapist

Health care professionals responsible for training body functions and activities

Rehabilitation

1. (General) A set of measures that assist individuals, who experience or are likely to experience disability, to achieve and maintain optimum functioning in interaction with their environments
https://www.who.int/news-room/fact-sheets/detail/rehabilitation

2. (Robotic) Mitigation and/or improvement of movement functions related to an impairment through body structures support or body functions replacement

3. Treatment to improve movement functions related to an impairment of a patient
(IEC 80601-2-78)

Notes:

- Rehabilitation can be carried out in situations where a patient has an impairment due to an accident or disease or congenital conditions (e.g. cerebral palsy) or can be used to slow down the expected loss of body functions due to neurodegenerative diseases (e.g. Parkinson's, multiple sclerosis).
- Rehabilitation is a general concept, that does not necessarily includes the use of robotic devices and does not only involve movement.

Related terms:

- Support
- Impairment

References:

IEC 80601-2-78 Particular requirements for basic safety and essential performance of medical robots for rehabilitation, assessment, compensation, or alleviation. 2008.

Robot control

Set of logic control and power functions which allows monitoring and control of the mechanical structure of the robot and communication with the environment (equipment and users)

References:

ISO 8373 Robots and robotic devices — Vocabulary, vol. 2012. 2008

Robot guidance

Directing of human motion along a trajectory by the application of external forces. (Moreno et al., 2013)

Notes:

- Industrial/Military field: the goal is to reduce the risk of (musculoskeletal) injury
- Rehabilitation field: the goal is to lead to a muscle coordination similar to the physiological one in order to promote motor recovery
- To not be confounded with robotic guidance in traditional industrial robotics:Robot motion following a predefined pattern by using sensory information (e.g. robotic arm in manufacturing/medical process).
- The guidance could be on Velocity, acceleration, jerk or torque profiles for example, among others

Related terms:

- Robot control
- Physical human-robot interaction
- Position control

References:

Moreno, J. C., Barroso, F., Farina, D., Gizzi, L., Santos, C., Molinari, M., & Pons, J. L. (2013). Effects of robotic guidance on the coordination of locomotion. *Journal of NeuroEngineering and Rehabilitation*, *10*(1). https://doi.org/10.1186/1743-0003-10-79

Robotic training

The repeated use of a robot to facilitate motor learning.

Notes:
This may apply for industry (e.g. procedures, posture), rehabilitation (recovery, enhancing function), medical (e.g. surgical skills), sport.

Related terms:

- Motor learning
- Human-robot interaction

Robustness

Correct functioning despite external and/or internal perturbations.

Notes:
A system can be robust while being unstable, and viceversa.

Semi-Active

(Semi-Active Actuator/Damper) Power mechanism that is active* during part of the work cycle which used to affect the motion of the wearable robot.

Notes:
*active means the addition of energy from an external source that is not part of the wearable robot mechanism.

Related terms:

- Semi-Passive
- Active
- Passive
- Quasi-Active
- Quasi-Passive.

Stability

Convergence to the desired state after external and/or internal disturbance

Notes:
A system can be robust while being unstable, and viceversa.

Related term:

- *Robustness*
- Coupled stability
- Core stability
- Joint stability

References:

Pollock, A., DURWARD, B., & ROWE, P. (2000). Pollock2000. *Clinical Rehabilitation, 2155*(December 1998), 402–406.

Committee, J., & Jcgm-wg, M. (2021). *International Vocabulary of Metrology Fourth edition – Committee Draft (VIM4 CD)*. (January), 1–55.

Stiffness

1. (Engineering) differential relation between infinitesimal differences in force and position which implies energy storage.
   SCORE: 3.889

2. Stiffness is the extent to which an object resists deformation in response to an applied force

   When linear force-displacement characteristic is applied, the stiffness can defined as:

   k = F / delta x = constant

   When the force-displacement relationship is nonlinear, stiffness vary with position as follows:

   k(x) = dF / dx /= constant

3. (Biomechanics) in active muscle, the total mechanical response defined by three components: muscle contraction, passive tissue and reflex response
   (Sinkjaer, Toft, Andreassen, & Hornemann, 1988)

References:

Sinkjaer, T., Toft, E., Andreassen, S., & Hornemann, B. C. (1988). Muscle stiffness in human ankle dorsiflexors: Intrinsic and reflex components. *Journal of Neurophysiology*, *60*(3), 1110–1121. https://doi.org/10.1152/jn.1988.60.3.1110

User interface

Means for information and action exchanges between human and robot during human–robot interaction

Notes:
Not all devices can be considered robotic

Related terms:
human–robot interaction

References:
ISO 8373 Robots and robotic devices — Vocabulary, vol. 2012. 2008.

Wearability

The ability of an exoskeleton (or garment) to be easily donned/doffed and to withstand prolonged wear comfortably (Gemperle, Kasabach, Stivoric, Bauer, & Martin, 1998)

Related terms:
Alignment

References:
Gemperle, F., Kasabach, C., Stivoric, J., Bauer, M., & Martin, R. (1998). Design for wearability. *International Symposium on Wearable Computers, Digest of Papers*, *1998-Octob*, 116–122. https://doi.org/10.1109/ISWC.1998.729537

Wearable robot

1. Device attached to the human body for assisting, enabling, or augmenting motor functions

2. (Exoskeleton) Device physically supporting a human and manipulating body parts through direct interaction and fixtures to a person, e.g. straps or clamps.

3. (Manipulator) Device providing fixture directly to a human without invasion, e.g. straps and clamps to provide direct interaction for dexterous manipulation.

Related terms:

- Assistance
- Enabling
- Augmentation
